# Prmt7 regulates the JAK/STAT/Socs3 signaling pathway in postmenopausal cardiomyopathy

**DOI:** 10.1038/s12276-024-01193-3

**Published:** 2024-03-14

**Authors:** Byeong-Yun Ahn, Yan Zhang, Shibo Wei, Yideul Jeong, Dong-Hyun Park, Sang-Jin Lee, Young-Eun Leem, Jong-Sun Kang

**Affiliations:** 1 Department of Molecular Cell Biology, Sungkyunkwan University, School of Medicine, Suwon, Republic of Korea; 2 Research Institute of Aging-Related Diseases, AniMusCure, Inc, Suwon, Republic of Korea

**Keywords:** Cardiac hypertrophy, Mechanisms of disease

## Abstract

Protein arginine methyltransferases (PRMTs) modulate diverse cellular processes, including stress responses. The present study explored the role of Prmt7 in protecting against menopause-associated cardiomyopathy. Mice with cardiac-specific Prmt7 ablation (cKO) exhibited sex-specific cardiomyopathy. Male cKO mice exhibited impaired cardiac function, myocardial hypertrophy, and interstitial fibrosis associated with increased oxidative stress. Interestingly, female cKO mice predominantly exhibited comparable phenotypes only after menopause or ovariectomy (OVX). Prmt7 inhibition in cardiomyocytes exacerbated doxorubicin (DOX)-induced oxidative stress and DNA double-strand breaks, along with apoptosis-related protein expression. Treatment with 17β-estradiol (E2) attenuated the DOX-induced decrease in Prmt7 expression in cardiomyocytes, and Prmt7 depletion abrogated the protective effect of E2 against DOX-induced cardiotoxicity. Transcriptome analysis of ovariectomized wild-type (WT) or cKO hearts and mechanical analysis of Prmt7-deficient cardiomyocytes demonstrated that Prmt7 is required for the control of the JAK/STAT signaling pathway by regulating the expression of suppressor of cytokine signaling 3 (Socs3), which is a negative feedback inhibitor of the JAK/STAT signaling pathway. These data indicate that Prmt7 has a sex-specific cardioprotective effect by regulating the JAK/STAT signaling pathway and, ultimately, may be a potential therapeutic tool for heart failure treatment depending on sex.

## Prmt7: The New Hope Against Gender-Specific Heart Failure

Heart disease, a major cause of death globally, affects men more than women. However, women after menopause are at greater risk than those before menopause. Scientists have discovered that the sex hormone E2 (a type of estrogen) helps protect against heart disease. In a recent study, a protein named Prmt7 was found to protect heart cells from heart failure. The study, which involved tests on mice and cell cultures (cells grown in a lab), showed that female mice without Prmt7 developed heart issues as they aged. The study also showed that Prmt7 is needed for E2 to fully function in heart cells. This suggests that Prmt7 could be a potential treatment target for heart failure, especially in women after menopause.

This summary was initially drafted using artificial intelligence, then revised and fact-checked by the author.

## Introduction

Diverse pathological conditions, such as hypertension, myocardial infarction, myocarditis, or heart valve disease, cause cardiac remodeling, leading to cardiomyocyte hypertrophy, fibrosis, necrosis, and eventually heart failure^1,2^. Interestingly, men are more susceptible to heart disease than women are; however, postmenopausal women are at greater risk than premenopausal women are for developing cardiovascular diseases, even though there is still a lower incidence of heart disease in postmenopausal women than in similarly aged men^3–5^.

Based on the discrepancy in the causes of cardiovascular disease according to sex and reproductive age, the sex hormone E2 is believed to be associated with protection against heart disease^5,6^. To date, multiple studies have been conducted to investigate the protective mechanism of E2 against cardiovascular diseases^6^. E2 enhances mitochondrial structure and function by activating the PI3K/ERK signaling pathway and reduces oxidative stress by promoting the generation of the antioxidants SOD2 and hydrogen sulfide^7^. Additionally, E2 is known to be involved in extracellular matrix (ECM) remodeling by modulating fibroblasts via estrogen receptors and the MAPK signaling pathway^8^. Additionally, E2 inhibits fibroblast proliferation and the expression of profibrotic genes, such as *Col1A1* (encoding Collagen I), *Col3A1* (encoding Collagen III), and *Fbrs* (encoding Fibrosin I), resulting in the suppression of fibrotic deposition, which is closely connected to hindering the relaxation and contraction of the heart^9^.

Protein arginine methylation is an important type of post-translational modification mediated by PRMTs and is commonly associated with normal cellular processes, including DNA repair, cell cycle regulation, transcription, mRNA splicing, and signal transduction^10,11^. PRMTs can be classified into three types according to their catalytic activity (I-III). The type I enzymes PRMT1, PRMT2, PRMT3, CARM1 (also known as PRMT4), PRMT6 and PRMT8 dimethylate their substrates asymmetrically. The type II enzymes PRMT5 and PRMT9 are symmetrical dimethyltransferases. The only member of the type III group is PRMT7, which can solely monomethylate arginine. Among them, Prmt7 has a unique structure consisting of two tandem PRMT modules, and there is growing information on the biological functions of Prmt7 in terms of gene expression, differentiation, senescence, and stress responses^12,13^.

In our previous study, we demonstrated that Prmt7 plays a protective role in cardiomyocytes against cardiac remodeling in heart failure^14^. Interestingly, during this study, cardiomyopathy resulting from Prmt7 deficiency was found only in young male mice and not in young female mice. This sex difference in the cardiomyopathy phenotype prompted us to question the distinct role of Prmt7 in female-related cardiac function. In this study, we found that cardiac-specific Prmt7 deficiency in postmenopausal or ovariectomized mice was associated with oxidative stress, apoptosis, and fibrosis, ultimately leading to severe cardiomyopathy. Additionally, there was a correlation between Prmt7 and E2, and Prmt7 was found to be required for the complete activation of E2 in cardiomyocytes. Analysis of the transcriptome and underlying mechanism demonstrated that Prmt7 plays a role in regulating the expression of Socs3, which functions as a negative feedback inhibitor in the JAK/STAT signaling pathway.

Taken together, our data have implications for understanding the cardioprotective role of Prmt7 in postmenopause-related cardiomyopathy and ultimately allow for the identification of new prognostic indicators or novel therapeutic strategies for the treatment of heart failure.

## Materials and methods

### Animal studies and echocardiography

*Prmt7*^*+/-*^ mice were maintained as previously described^15^. Briefly, C57BL/6N-Tyr^c-Brd^ *Prmt7*^tm1a (EUCOMM) wtsi^/WtsiCnbc mice purchased from the Sanger Institute were backcrossed onto a C57BL/6 J background for at least 10 generations. Littermate wild-type mice were used as control groups for *Prmt7*^*-/-*^ mice in all the experiments. For generation of cardiac-specific *Prmt7* null mice, *Prmt7*^*Tm1c/Tm1c*^ (*Prmt7*^*f/f*^) mice were crossbred with mice harboring the *Myh6-Cre* transgene [Tg(Myh6-cre)2182Mds/J] (Jackson Laboratory, Bar Harbor, ME). Genotyping was performed as previously described^15^. To assess the effect of Prmt7 on cardiomyopathy after menopause, we utilized 10-month-old female littermates obtained through heterozygous breeding. For the development of OVX, the ovaries of 2-month-old female mice were surgically excised, and 7 days later, 25 mg/kg DOX (Tocris Bioscience, Bristol, UK) was administered to induce cardiotoxicity. The mice were subjected to echocardiography 4 days after administration and were sacrificed the next day.

For echocardiographic analysis, the mice were anesthetized with 1–2% (vol/vol) isoflurane. Echocardiography was performed one day before the mice were sacrificed using a Vevo LAZR-X photoacoustic imaging system (Fujifilm VisualSonics, Toronto, Canada). Heart rate was monitored and generally maintained at 400-500 beats per minute. Analyses of M-mode images derived from the short-axis view of the left ventricle (LV) were performed to calculate the ejection fraction (EF) and fractional shortening (FS).

For animal studies, drug administration was approved by the Institutional Animal Care and Use Committee (IACUC) of the Sungkyunkwan University School of Medicine (SUSM) and carried out according to ethical guidelines.

### Cell culture and transfection

H9c2 cells were cultured in Dulbecco’s modified Eagle’s medium (DMEM; Gibco, Billings, MT) supplemented with 10% fetal bovine serum (FBS; Gibco, Billings, MT) and 1% penicillin/streptomycin (Thermo Fisher Scientific, Waltham, MA). Newborn rat ventricular myocytes (NRVMs) were isolated from neonatal Sprague–Dawley (SD, 1–2 days) rat heart tissues as previously described^14^. For analysis of the correlation between Prmt7 and E2 under cardiotoxic conditions, H9c2 cells were exposed to 10 nM E2 (Sigma‒Aldrich) and/or 1 μM DOX for 16 h. Transfection studies were performed as previously described^14^. Polyethylenimine (1 mg/ml; Sigma‒Aldrich, St. Louis, MO) was used to transfect pcDNA-PRMT7 or pcDNA-HA-PRMT7 into H9c2 cells. These overexpression plasmids encode human PRMT7^15^. For Prmt7 depletion, a lentiviral vector containing either shScrambled or shPrmt7 was utilized, as previously described^16^. For analysis of the effects of Prmt7 inhibition, cells were treated with 50 μM DS-437 (Sigma‒Aldrich), a dual inhibitor of Prmt5 and Prmt7, or 1 μM SGC-8158 (Sigma‒Aldrich) for 24 h. For activation of the JAK/STAT signaling pathway, H9c2 cells were treated with 10^4^ units/ml of LIF (Sigma‒Aldrich) for 30 min.

### Protein and RNA analysis

Immunoblotting and immunostaining were performed as previously described^14^. Briefly, cultured cells were lysed using lysis buffer (50 mM Tris-HCl (pH 7.4), 1.5 mM MgCl_2_, 150 mM NaCl, 1 mM EGTA, 1% Triton X-100, and complete protease inhibitor cocktail) and analyzed by standard western blotting. The antibodies used in this study are as follows: Prmt7 (Abcam, ab179822, 1:500 dilution), cleaved Caspase-3 (Cell Signaling, Cat# 9664, 1:500 dilution), γH2AX (Cell Signaling, Cat# 9718, 1:500 dilution), HA (Cell Signaling, Cat# 3724, 1:1000 dilution), β-actin (Cell Signaling, #4970, 1:500 dilution), pSTAT3 (Cell Signaling, Cat# 9145, 1:500 dilution), STAT3 (Cell Signaling, Cat# 9139, 1:500 dilution), and HSP90 (Santa Cruz, sc-7947, 1:1000 dilution). The quantification of protein levels was performed by signal density analysis using the ImageJ program, after which the protein levels were normalized to the level of a loading control, such as β-actin or HSP90.

Quantitative RT‒PCR was performed as previously described^14^. Total RNA from mouse hearts was extracted with easy-BLUE reagent (iNtRON, Seongnam, Republic of Korea) following the manufacturers’ instructions. cDNAs were synthesized from 0.5 μg of total RNA by using a PrimeScript RT reagent kit (TaKaRa, Shiga, Japan) according to the manufacturer’s instructions. Gene expression was quantified by using SYBR Premix Ex Taq (TaKaRa) on a Thermal Cycler Dice Real-Time System (TaKaRa, TP800) following the manufacturer’s instructions. The sequences of primers used in this study are indicated below; *IL-1α* (Forward) 5’-GCCCGTGTTGCTGAAGGAGT-3’ (Reverse) 5′-CATAGAGGGCAGTCCCCGTG-3′; *IL-6* (Forward) 5′-TACCACTTCACAAGTCGGAGGC-3′ (Reverse) 5′-CTGCAAGTGCATCATCGTTGTTC-3′; *IL-18* (Forward) 5′-GACAGCCTGTGTTCGAGGATATG-3′ (Reverse) 5′-TGTTCTTACAGGAGAGGGTAGAC-3′; *IFN-γ* (Forward) 5′-CAGCAACAGCAAGGCGAAAAAGG-3′ (Reverse) 5′-TTTCCGCTTCCTGAGGCTGGAT-3′; *TNF-α* (Forward) 5′-AAATGGGCTCCCTCTCATCAGTTC-3′ (Reverse) 5′- TCTGCTTGGTGGTTTGCTACGAC-3′; *Socs3* (Forward) 5′-GACCTGTCTACAGCTCTCCGTC-3′ (Reverse) 5′-CTGCGCCTCCTATGGTCCC-3′; *18S rRNA* (Forward) 5′-GTAACCCGTTGAACCCCATT-3′ (Reverse) 5′-CCATCCAATCGGTAGTAGCG-3′; and *L-32* (Forward) 5′-GGCCTCTGGTGAAGCCCAAGATCG-3′ (Reverse) 5′-CCTCTGGGTTTCCGCCAGTTTCGC-3′.

The transcriptome of *PRMT7*-expressing heart tissue from young and old human males and females was analyzed with the GSE36961 dataset from the Gene Expression Omnibus (GEO; http://www.ncbi.nlm.nih.gov/geo).

High-throughput sequencing was performed as single-end 75 sequencing using an Illumina NExtSeq 500 (Ebiogen, Seoul, Korea). The analysis of RNA sequencing data was performed by using ExDEGA v3.0 (Ebiogen) and Morpheus (http://software.broadinstitute.org/morpheus/). Global gene expression was assessed via Reactome with gene set enrichment analysis (GSEA) (http://www.gsea-msigdb.orb/gsea/msigdb/index.jsp) using the MsigDB database v7.2 (>1.3-fold, RC log2 > 1.0, *P* < 0.1).

### Immunohistochemistry and immunofluorescence

Immunohistochemistry of heart sections was performed as previously described^17^. Briefly, the harvested mouse hearts were fixed with 4% paraformaldehyde (PFA) and then embedded in either a paraffin block or optimal cutting temperature (OCT) solution (Sakura Finetek, USA; Torrance, CA). The embedded heart samples were sectioned to 5-7 μm thickness and stained using an anti-wheat germ agglutinin (WGA) antibody (Abcam, ab20528), a Trichrome stain kit (Abcam), or a Picro Sirius Red stain kit (Abcam). The presence of intracellular reactive oxygen species (ROS) was detected using dihydrorhodamine-123 (DHR-123; Cayman Chemical). The samples were incubated with 5 μM DHR-123 at 37 °C for 15 min according to the manufacturer’s instructions. Nuclei were stained with Hoechst 33342 (Invitrogen). Images were captured with a Nikon ECLIPS TE-2000U (Nikon, Tokyo, Japan) and analyzed with NIS-Element F software (Nikon) or ImageJ.

Immunofluorescence was performed as previously described^14^. Briefly, cells were fixed with 4% PFA, permeabilized with 0.2% Triton X-100 in PBS, and blocked (2% BSA or 5% goat serum in PBS). For γH2AX staining, anti-phospho-histone H2A.X antibody (Cell Signaling Technology, Cat# 9718, 1:250 dilution) was used. Intracellular ROS were detected by incubating cells with 5 μM DHR-123 at 37 °C for 15 min following the manufacturer’s instructions. Nuclei were counterstained with Hoechst 33342 (Invitrogen). The fluorescence images were analyzed with an LSM-710 confocal microscope (Carl Zeiss) and processed with either ZEN software (Carl Zeiss) or ImageJ software.

### Chromatin immunoprecipitation assay

Chromatin immunoprecipitation (ChIP) analysis was performed as previously described^17^. H9c2 cells that were treated with DMSO or 1 μM SGC-8158 for 24 h were crosslinked with 0.75% formaldehyde, and the reaction was quenched with 125 mM glycine. After sonication in ChIP lysis buffer (50 mM HEPES-KOH (pH 7.5), 140 mM NaCl, 1 mM EDTA (pH 8.0), 1% Triton X-100, 0.1% sodium deoxycholate, 0.1% SDS, and complete protease inhibitor cocktail), the chromatin samples were immunoreacted with anti-STAT3 (Cell Signaling Technology, Cat# 9139), anti-acetyl H4K8 (Cell Signaling Technology, #2594), anti-histone H3 (trimethyl K27) (Abcam, ab6002), or an isotype IgG antibody, and salmon sperm DNA-adsorbed protein A agarose (Merck Millipore, Burlington, MA). After washing and eluting, the STAT3-binding region in the *Socs3* promoter^18,19^ was amplified with the forward primer 5′- CACAGCCTTTCAGTGCAGAG-3′ and the reverse primer 5′- AGAGACAGCGGTGGCAAG-3′. The enrichment of the genes was quantified via qPCR with SYBR premix EX Taq (TaKaRa) on a Thermal Cycler Dice Real Time System (TaKaRa, TP800) following the manufacturer’s instructions.

### Statistical analysis

The values are presented as the means ± SEMs or SDs. Statistical significance was calculated by paired or unpaired two-tailed Student’s *t* test or analysis of variance (ANOVA) test followed by Tukey’s test; differences were considered significant at *P < 0.05, ** *P* < 0.01, and *** *P* < 0.005.

## Results

### Young female cKO mice are less susceptible to cardiomyopathy than male cKO mice

**Fig. 1:**
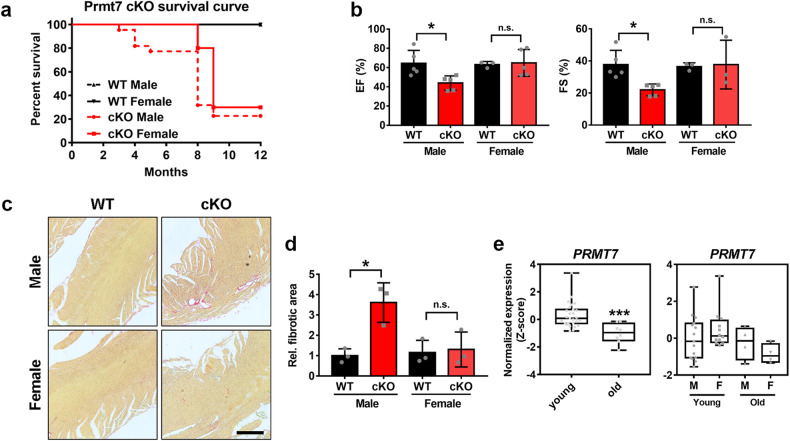
Young female mice with Prmt7 ablation in cardiomyocytes are less susceptible to the development of cardiac dysfunction and fibrosis than male mice. **a** Survival rates of male and female WT and cKO mice from postnatal day 1 to 12 months (WT male (*n* = 10), WT female (*n* = 10), cKO male (*n* = 22), and cKO female (*n* = 10)). **b** Echocardiographic parameters of male and female WT and cKO mice at 3 months after delivery. EF (male, WT = 5, cKO = 5; female, WT = 3, cKO = 5) and FS (male, WT = 5, cKO = 5; female, WT = 3, cKO=3) *P < 0.05, n.s.= not significant; one-way ANOVA. The data represent the mean ± SEM. **c** Representative images of Sirius Red staining of the hearts of male and female WT and cKO mice at 3 months after delivery. Scale bar = 100 μm. **d** Quantification of the fibrotic area in whole heart areas (*n* = 3, each) as shown in panel **c**. *P < 0.05, n.s.= not significant; one-way ANOVA. The data represent the mean ± SEM. **e** Boxplots showing the expression of *PRMT7* in young (4–49 years old; male, *n* = 15; female, *n* = 12) and old (51–65 years old: male, *n* = 4; female, *n* = 4) human hearts (GSE36961). ***P < 0.005.

In our previous study on the role of Prmt7 in cardiac function, we showed that cardiac-specific Prmt7 ablation resulted in cardiac hypertrophy accompanied by fibrosis^14^. One interesting aspect of cardiac-specific Prmt7-ablated mice was the sex difference in their cardiomyopathy phenotype and survival. We initially analyzed the difference in survival probability between male and female WT and cKO mice (Fig. 1a). Interestingly, male cKO mice started to die at ~3 months of age, while female cKO mice exhibited dramatic lethality at 8 months of age. Thus, we examined the detailed phenotype of female mice lacking cardiac Prmt7 and the underlying mechanisms for the sex differences. When we performed echocardiography analysis of 3-month-old male and female mice, male cKO mice exhibited decreases in EF and FS, but these changes were not observed in female cKO mice (Fig. 1b). Additionally, the severe fibrosis detected in male cKO cardiac tissue was not observed in female cKO hearts (Fig. 1c, d). Furthermore, in an analysis of datasets obtained from human cardiac tissue (GSE36961), the expression of *PRMT7* was significantly downregulated in cardiac tissues from elderly individuals (Fig. 1e). When these data were further analyzed for sex differences, the expression of *PRMT7* was found to be similar in young and old male hearts. However, a decrease in *PRMT7* expression was significantly greater in the cardiac tissues of aged females than in those of young females, indicating that there is a close correlation between PRMT7 and aging in female hearts. Taken together, these data suggest that Prmt7 is involved in a distinct regulatory mechanism for cardiac function in females that depends on aging.

### Aged female cKO mice exhibit cardiac hypertrophy accompanied by fibrosis and oxidative stress

**Fig. 2:**
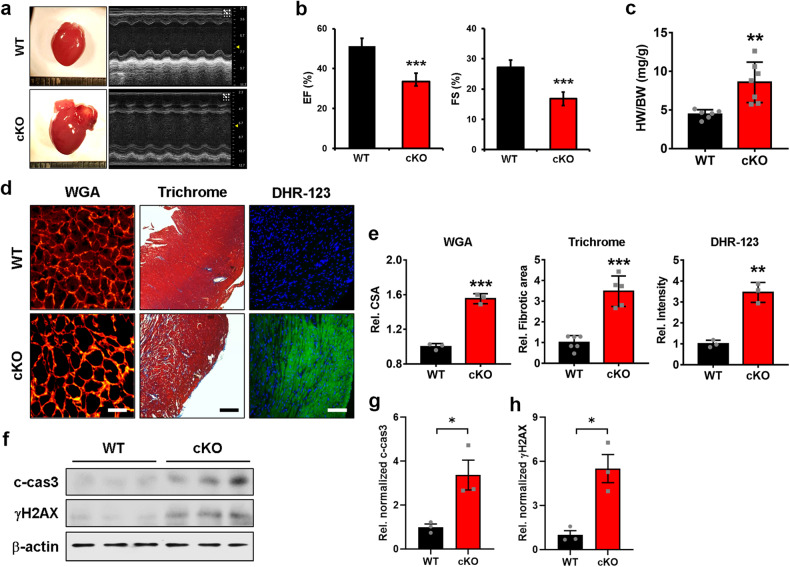
Aged female mice lacking Prmt7 exhibit cardiac hypertrophy accompanied by interstitial fibrosis and oxidative stress. **a** Photograph of hearts isolated from 10-month-old female mice, both WT and cKO. Representative echocardiographic images of female WT and cKO mice 1 day before heart isolation. **b** Echocardiographic parameters (EF and FS) of female WT (*n* = 6) and cKO (*n* = 7) mice. ***P < 0.005. The data are presented as the means ± SEMs and were analyzed by ANOVA. **c** The relative heart weights (HWs) of female WT (*n* = 6) and cKO (*n* = 7) mice were normalized to body weight (BW). **P < 0.01. The data are presented as the means ± SDs. **d** Representative images of WGA staining, Masson’s trichrome staining, and DHR-123 staining of female WT and cKO hearts. Scale bar = 50 µm (Left), 100 µm (Center and Right). **e** Quantification of the cross-sectional area (CSA) (*n* = 3 per group), fibrotic area in the whole heart (WT, *n* = 6; cKO, *n* = 5), and relative fluorescence intensity (*n* = 3 per group) are shown in panel **d**. **P < 0.01, ***P < 0.005. The data represent the mean ± SEM. **f** Immunoblotting analysis of c-cas3 and γH2AX in female WT and cKO hearts. **g**, **h** Quantification of the protein levels of c-cas3 (**g**) and γH2AX (**h**) are shown in panel **f**. *P < 0.05. The data represent the mean ± SD.

To gain insight into the effect of Prmt7 deficiency in aged female mice, we examined the cardiac function of female WT and cKO mice at the age of 10 months, when high lethality was observed in female cKO mice, by performing echocardiographic analysis. Compared to the WT mice, the female cKO mice exhibited cardiac dysfunction, as evidenced by the decreased EF and FS (Fig. 2a, b). Additionally, compared with those of WT mice, the relative heart weights of cKO mice were significantly greater (Fig. 2c). WGA and Masson’s trichrome staining of female WT and cKO hearts demonstrated that, compared with WT hearts, cKO hearts had enlarged cardiomyocytes and more collagen lesions (Fig. 2d, e). Furthermore, evaluation of ROS generation using the DHR-123 fluorescent probe showed increased production of ROS in cKO hearts relative to WT hearts (Fig. 2d, e). Immunoblotting against c-cas3 and γH2AX also demonstrated that Prmt7 deficiency in the heart led to an increase in cellular apoptosis and DNA damage in aged females (Fig. 2f–h). These data suggest that female mice with cardiac-specific Prmt7 deficiency develop late-onset cardiac hypertrophy accompanied by increased interstitial fibrosis and oxidative stress.

### Ovariectomized female cKO mice are more vulnerable to DOX-induced cardiomyopathy

**Fig. 3:**
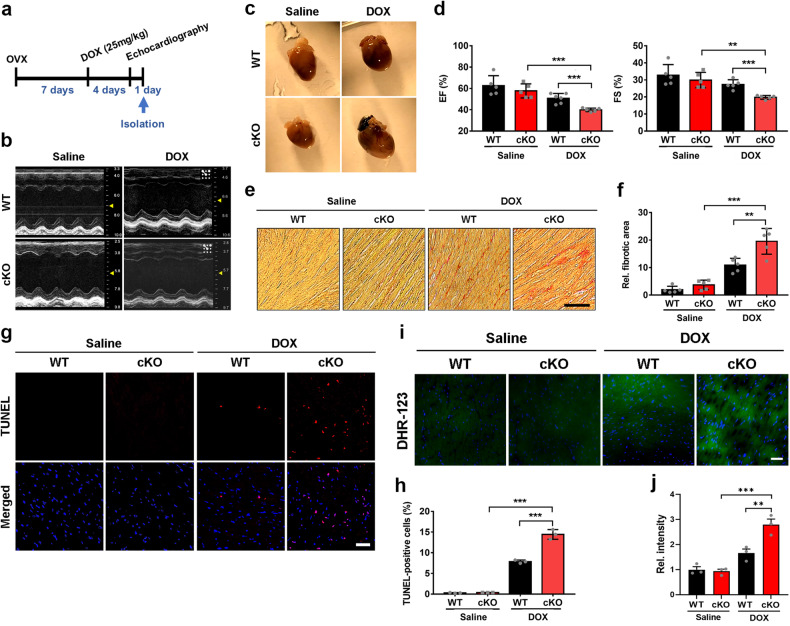
OVX-induced young female cKO mice are more vulnerable to DOX-induced cardiomyopathy. **a** Experimental scheme for ovariectomized mice. Two-month-old female mice were subjected to OVX and administered DOX after 7 days. After 4 days, echocardiographic analysis was performed, and the mice were subsequently sacrificed. **b** Representative echocardiographic images of WT-OVX and cKO-OVX mice that were administered saline or DOX (*n* = 5 per group). **c** Photograph of hearts isolated from WT-OVX and cKO-OVX mice that were administered saline or DOX. **d** The echocardiographic parameters (EF and FS) of WT-OVX and cKO-OVX mice that were administered saline or DOX (*n* = 5 per group). **P < 0.01, ***P < 0.005; one-way ANOVA. The data represent the mean ± SEM. **e** Representative images of Sirius Red-stained sections of WT-OVX and cKO-OVX mice that were administered saline or DOX. Scale bar = 100 μm. **f** Quantification of the fibrotic area in whole heart areas (*n* = 5) is shown in panel **e**. **P < 0.01, ***P < 0.005, one-way ANOVA. The data represent the mean ± SEM. **g** Representative confocal microscopy images of TUNEL-positive nuclei (red) in the hearts of WT-OVX and cKO-OVX mice that were administered saline or DOX. Scale bar = 50 μm. **h** Quantification of TUNEL-positive nuclei as shown in panel **g** (*n* = 3 per group). ***P < 0.005. The data are presented as the means ± SEMs and were analyzed by ANOVA. **i** Representative confocal microscopy images of DHR-123 (green) in the hearts of WT-OVX and cKO-OVX mice that were administered saline or DOX. Scale bar = 50 μm. **j** The quantification of the relative fluorescence intensity is shown in panel **i** (*n* = 4 per group). **P < 0.01, ***P < 0.005. The data are presented as the means ± SEMs.

The onset of cardiac hypertrophy in female cKO mice at the age of 9–10 months, when reproductive senescence transition occurs, prompted us to investigate the effect of Prmt7 deficiency by employing a surgical menopausal model. Two-month-old female mice, both WT and cKO, were subjected to OVX, and 1 week later, DOX was administered for 5 days to induce virulent cardiotoxicity. One day before heart excision, echocardiography was performed to assess cardiac function (Fig. 3a). The depletion of cardiac-specific Prmt7 resulted in a reduced EF and FS following surgical menopause, and the administration of DOX exacerbated the cardiac dysfunction caused by Prmt7 deficiency (Fig. 3b–d). Sirius Red staining of ovariectomized WT and cKO hearts revealed that cardiac Prmt7 depletion was associated with fibrosis, and this increase in fibrosis severity was more pronounced in the presence of DOX (Fig. 3e, f). Furthermore, in the presence of DOX, the cKO hearts exhibited a more substantial increase in cell death than the WT hearts did (Fig. 3g, h). DHR-123 staining for measuring ROS production also showed that Prmt7 depletion elevated ROS levels compared to those of the WT group under DOX stimulation (Fig. 3i, j). Taken together, these findings showed that ovariectomized cKO mice exhibited aggravated cardiomyopathy and cardiotoxicity following DOX administration.

### Prmt7 protects cardiomyocytes from DOX-induced DNA damage and cell death

**Fig. 4:**
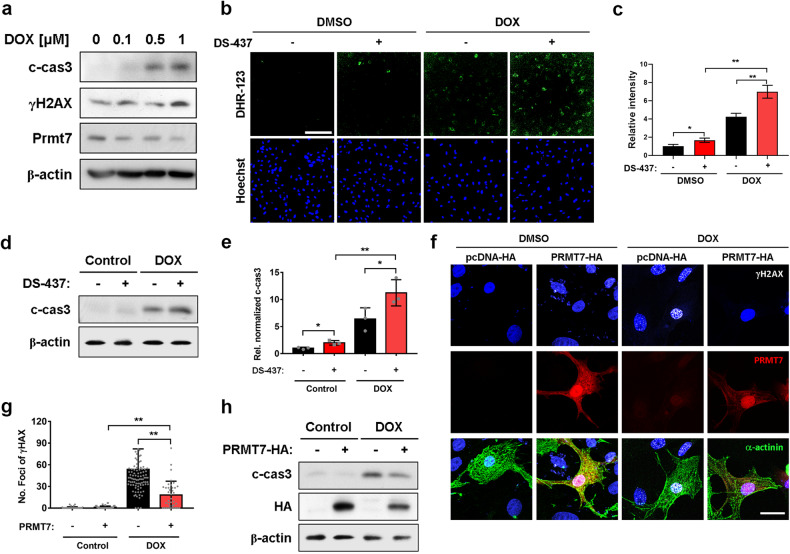
Prmt7 protects cardiomyocytes from DOX-induced DNA damage and cell death. **a** Immunoblot analysis of H9c2 cells treated with DOX. **b** Representative confocal microscopy images of DHR-123 (green) in H9c2 cells cultured with vehicle, DS-437 or DOX for 24 h. Scale bar = 100 μm. **c** Quantification of the relative fluorescence intensity is shown in panel **b**. *P < 0.05, **P < 0.01. The data represent the mean ± SEM. **d** Immunoblot analysis of DOX-treated H9c2 cells cultured with vehicle or DS-437 for 24 h. **e** Quantification of c-cas3 expression in DOX-treated H9c2 cells cultured with vehicle or DS-437, as shown in panel **d**. *P < 0.05, **P < 0.01, one-way ANOVA. The data represent the mean ± SD. **f** Representative confocal microscopy images of γH2AX (white) and PRMT7 (red) in DMSO- or DOX-treated NRVMs transfected with pcDNA-HA or pcDNA-PRMT7-HA. Nuclei were visualized with DAPI (blue), and the cytoskeleton was stained with α-actinin (green). Scale bar = 10 μm. **g** The quantification of the relative fluorescence intensity is shown in panel **f**. **P < 0.01, one-way ANOVA. The data represent the mean ± SEM. **h** Immunoblot analysis of c-cas3 and HA in DOX-treated H9c2 cells transfected with pcDNA-PRMT7-HA. β–actin was used as a loading control.

Oxidative stress and apoptosis are factors contributing to the pathogenesis of menopause^20,21^. Moreover, DOX is a well-known drug that can cause cardiomyopathy as a side effect and is associated with oxidative stress and apoptosis^22,23^. Therefore, we investigated the role of Prmt7 in oxidative stress and apoptosis in cardiomyocytes by utilizing DOX. For this purpose, H9c2 cardiomyocytes were treated with different amounts of DOX, and the expression of Prmt7 was examined (Fig. 4a). As the concentration of DOX increased, the levels of cleaved caspase 3 and γH2AX increased, while the level of Prmt7 decreased, indicating a negative correlation between Prmt7 and DOX-induced cardiotoxicity. Then, we pharmacologically inhibited Prmt7 activity in H9c2 cells by treating them with DS-437 and assessed the effect of this inhibition on DOX-induced oxidative stress using the ROS indicator DHR-123 (Fig. 4b, c). When Prmt7 activity was inhibited during DOX exposure, ROS generation increased compared to that in the vehicle treatment group. Analysis of apoptosis also showed that inhibiting Prmt7 activity led to an increase in the level of cleaved caspase 3, which was further elevated by DOX treatment (Fig. 4d, e). Next, we investigated the effect of Prmt7 on DOX-induced DNA damage and apoptosis in NRVMs via lentiviral vector-mediated PRMT7 overexpression. The γH2AX staining results demonstrated that cells overexpressing PRMT7 presented a decreased number of γH2AX foci compared to cells without PRMT7 overexpression after exposure to DOX (Figs. 4f, g). In addition, PRMT7 overexpression led to a dramatic reduction in the level of cleaved caspase 3 during DOX treatment, supporting the recovery effect of PRMT7 overexpression on DOX-induced cardiotoxicity (Fig. 4h). These findings suggest that Prmt7 has a protective effect on DOX-induced cardiomyocyte death by preventing oxidative stress and apoptosis.

### Prmt7 is required for estrogen-mediated protection of cardiomyocytes

**Fig. 5:**
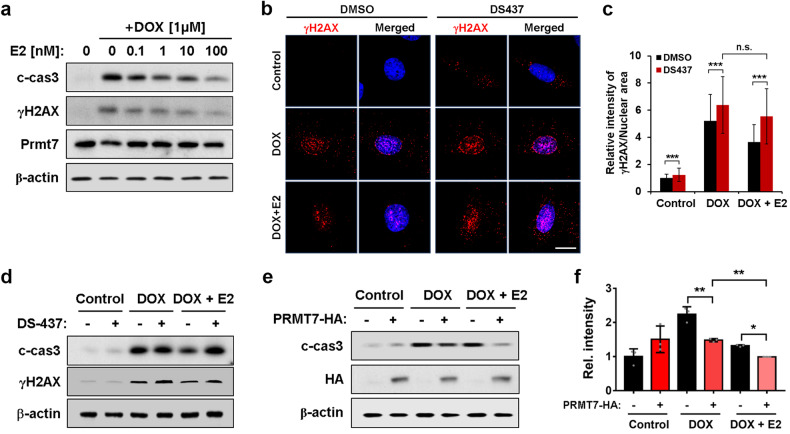
Prmt7 is required for estrogen-mediated protection of cardiomyocytes. **a** Immunoblot analysis of H9c2 cells treated with DOX and various concentrations of E2. **b** Representative confocal microscopy images of γH2AX (red) in H9c2 cells incubated with DOX in combination with E2 and/or DS-437 for 16 h. Scale bar = 10 μm. **c** Quantification of the relative fluorescence intensity of γH2AX is shown in panel **b**. ***p < 0.005, n.s. not significant; one-way ANOVA. The data represent the mean ± SEM. **d** Immunoblot analysis of DS-437-treated H9c2 cells incubated with DOX and/or E2 for 16 h. **e** Immunoblot analysis of pcDNA-PRMT7-HA-transfected H9c2 cells that were incubated with DOX and/or E2 for 16 h. **f** Quantification of c-cas3 levels in H9c2 cells transfected with pcDNA-PRMT7-HA and incubated with DOX and/or E2, as shown in panel **e**. *P < 0.05, **P < 0.01, one-way ANOVA. The data represent the mean ± SD.

Considering that estrogen depletion is a critical contributor to cardiomyopathy in aging females, we investigated the correlation between Prmt7 and estrogen in H9c2 cells with cardiotoxicity. H9c2 cells were treated with 1 μM DOX in combination with increasing concentrations of E2 (Fig. 5a). E2 treatment caused a dose-dependent reduction in the levels of cleaved caspase 3 and γH2AX even in the presence of DOX. Interestingly, the expression of Prmt7, which was reduced by DOX, was restored by E2 treatment. For verification of the role of Prmt7 in E2-mediated protection of cardiomyocytes, H9c2 cells were treated with DOX in combination with E2 and/or DS-437 and then subjected to γH2AX immunostaining (Fig. 5b, c). The inhibition of Prmt7 activity by DS-437 was associated with a severe increase in the formation of DOX-induced γH2AX foci. However, contrary to our expectations, the addition of E2 barely reversed DNA damage when Prmt7 activity was lost. Consistently, the suppressive effect of E2 on the level of cleaved caspase 3 was not significant when Prmt7 was inhibited (Fig. 5d). In contrast, the attenuation of cleaved caspase 3 levels, which was driven by the ectopic expression of PRMT7, was further intensified when combined with E2 administration under DOX conditions (Fig. 5e, f). Taken together, these data suggest that there is a correlation between Prmt7 and estrogen and that Prmt7 is required for the full cardioprotective effects of E2.

### Prmt7-deficient hearts of ovariectomized mice exhibit altered gene expression profiles associated with the JAK/STAT signaling pathway

**Fig. 6:**
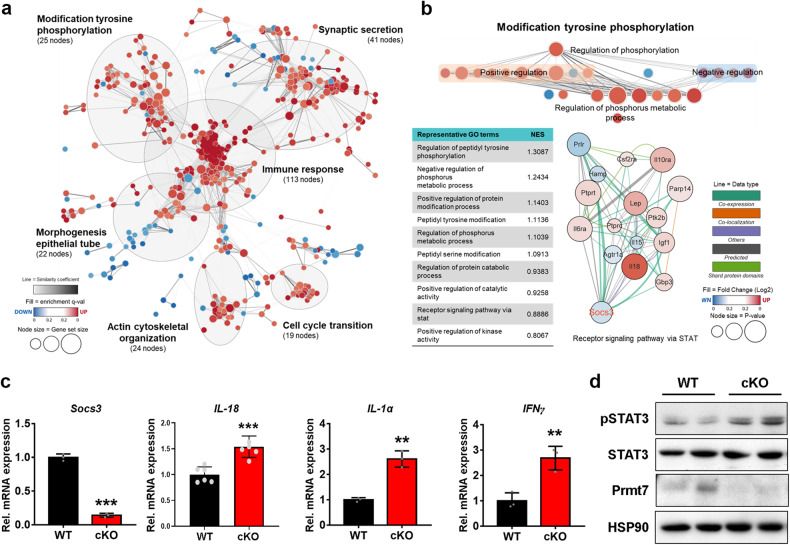
Prmt7-deficient hearts of OVX-induced mice exhibit altered gene expression profiles associated with the JAK/STAT signaling pathway. **a** Enrichment map comparing Gene Ontology (GO) terms between WT and cKO mice. The nodes represent gene sets connected by edges, grouped in subclusters, and manually annotated. **b** Representative GO terms of subclusters related to “response to stress” from panel **a**. A gene interaction network was constructed from “response to stress” gene sets, with different lines indicating interaction types. **a**, **b** Fold change (FC) > 1.3, normalized data (log2) > 1.0 and *P* value cutoff < 0.1 according to Student’s *t* test. The genes were initially analyzed using GSEA, followed by enrichment map analysis using Cytoscape. **c** qRT‒PCR analysis of the expression levels of genes associated with the JAK/STAT signaling pathway in hearts from WT and cKO mice (*n* = 3). **P < 0.01, ***P < 0.005. The data represent the mean ± SD. **d** Immunoblot analysis of hearts from WT and cKO mice.

To investigate the mechanism underlying female cKO-associated cardiomyopathy, we performed RNA sequencing of hearts from ovariectomized WT and cKO mice, followed by GSEA (>1.3-fold, average of the normalized RC log2 > 1.0, and *P* value < 0.1). Analysis of the data indicated that the expression of genes associated with the immune response, tyrosine phosphorylation, synaptic secretion, and cell cycle transition was significantly altered in cKO hearts compared to WT hearts (Fig. 6a). Among others, the subcluster “modification of tyrosine phosphorylation” related to the stress response was further analyzed based on the gene interaction network (Fig. 6b). The results revealed alterations in the expression of genes that regulate the receptor signaling pathway via STAT in cKO hearts compared to WT hearts. The JAK/STAT signaling pathway is a signaling cascade of various proinflammatory cytokines that is known to be involved in inflammatory responses and apoptosis^24,25^. In cKO hearts, the expression of *Socs3*, which is a key feedback inhibitor that negatively regulates the JAK/STAT signaling pathway, was noticeably decreased. However, the levels of *IL-18*, which is a cytokine known for its critical involvement in autoimmune and inflammatory diseases, were elevated. To validate the changes in expression in cKO mice, we measured the levels of *Socs3*, *IL-18*, *IL-1α,* and *IFN-γ* by performing qRT‒PCR on total RNA extracted from the hearts of ovariectomized WT and cKO mice. Similar to the previous RNA sequencing data, in the present study, the cKO hearts exhibited a significant reduction in the level of *Socs3*, whereas the levels of *IL-18*, *IL-1α*, and *IFN-γ* were substantially greater than those in the WT hearts (Fig. 6c). Furthermore, when we assessed the activity of the JAK/STAT signaling pathway by determining the level of pSTAT3/STAT3, cKO hearts exhibited elevated pSTAT3 levels compared to those in WT hearts (Fig. 6d). Taken together, these data suggest that Prmt7 deficiency results in dysregulation of the JAK/STAT signaling pathway, which likely contributes to increased inflammation and related cellular stress in the hearts of ovariectomized female mice.

### Prmt7 regulates the activation of the JAK/STAT signaling pathway through Socs3

**Fig. 7:**
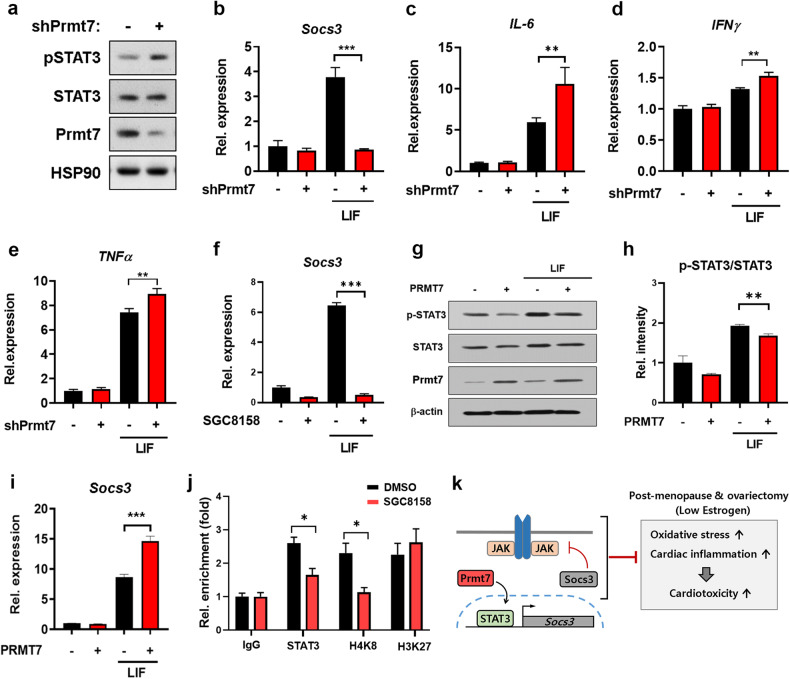
Prmt7 regulates the activity of the JAK/STAT signaling pathway through Socs3. **a** Immunoblot analysis of H9c2 cells infected with shScrambled- or shPrmt7-expressing lentivirus and then treated with LIF for 30 minutes. **b**–**e** qRT‒PCR analysis of the levels of *Socs3* (**b**), *IL-6* (**c**), *IFNγ* (**d**), and *TNFα* (**e**) in Prmt7-depleted H9c2 cells treated with either vehicle or LIF. **P < 0.01, ***P < 0.005; one-way ANOVA. The data represent the mean ± SD. **f** qRT‒PCR analysis of *Socs3* expression in DMSO- or SGC8158-treated H9c2 cells treated with LIF for 30 minutes. ***P < 0.005, one-way ANOVA. The data represent the mean ± SD. **g** Immunoblot analysis of PRMT7-overexpressing H9c2 cells treated with vehicle or LIF for 30 min. **h** Quantification of p-STAT3 levels, which were normalized to the level of STAT3 in LIF-treated PRMT7-overexpressing H9c2 cells, as shown in panel **g**. **P < 0.01, one-way ANOVA. The data represent the mean ± SD. **i** qRT‒PCR analysis of *Socs3* levels in LIF-treated PRMT7-overexpressing H9c2 cells. ***P < 0.005, one-way ANOVA. The data represent the mean ± SD. **j** ChIP assay to determine the enrichment of STAT3 and histone modifications in the *Socs3* promoter in DMSO- or SGC8158-treated H9c2 cells after exposure to LIF for 30 min. *P < 0.05, one-way ANOVA. The data represent the mean ± SD. **k** A scheme describing the function of Prmt7 in the regulation of the JAK/STAT signaling pathway under postmenopausal and ovariectomized conditions.

For analysis of the role of Prmt7 in the regulation of the JAK/STAT signaling pathway, H9c2 cells were infected with shScrambled- or shPrmt7-expressing lentivirus and subjected to immunoblotting for STAT3 phosphorylation. Similar to the results obtained with cKO hearts, Prmt7-depleted cells showed activation of the JAK/STAT signaling pathway, as evidenced by the increase in the phosphorylation of STAT3 compared to that in control cells (Fig. 7a). In addition, Prmt7 depletion attenuated the expression of *Socs3* (Fig. 7b) but significantly upregulated the expression of *IL-6*, *IFNγ*, and *TNFα* during LIF-induced activation of the JAK/STAT3 cascade (Fig. 7c–e). To confirm these results, we selectively suppressed the enzymatic activity of Prmt7 using the Prmt7-specific inhibitor SGC8158 and determined the transcription of *Socs3* by qRT‒PCR. The results showed that inhibiting Prmt7 activity also had a negative impact on the expression of *Socs3* (Fig. 7f). Conversely, ectopic expression of PRMT7 attenuated the activation of the JAK/STAT pathway, as indicated by a decrease in the p-STAT3/STAT ratio (Fig. 7g, h), while concurrently increasing the level of the negative regulator Socs3 (Fig. 7i). To elaborate on the mechanism by which Prmt7 regulates JAK/STAT signaling-mediated inflammation, we initially selected Socs3 as a key target of Prmt7 for the control of the JAK/STAT signaling pathway. To investigate this phenomenon, we asked whether Prmt7 is involved in STAT3-mediated Socs3 expression. DMSO- or SGC8158-treated H9c2 cells were exposed to LIF for 30 min to activate the JAK/STAT3 signaling pathway, and the recruitment of STAT3 to the *Socs3* promoter was analyzed via a ChIP assay (Fig. 7j). When Prmt7 activity was repressed by SGC8158, the STAT3-binding region of the *Socs3* promoter was less occupied by STAT3 than was the control. Additionally, restricted chromatin accessibility to the *Socs3* promoter was indicated by decreased enrichment of acetylated H4K8 and increased enrichment of methylated H3K27 in the *Socs3* promoter. Taken together, these data suggest that Prmt7 is critical for regulating the JAK/STAT3 signaling pathway through the mediation of Socs3 expression in cardiomyocytes.

## Discussion

A previous study revealed that Prmt7 deficiency in the heart is linked to cardiomyopathy^14^. In the present study, we found that the effects of Prmt7 deficiency on the heart differ according to sex and age. Unlike male mice, young female mice rarely exhibit any symptoms of cardiac disease when Prmt7 is specifically depleted in the heart. Moreover, cardiomyopathy is strongly observed in aging female mice during menopause or after surgical removal of ovaries. This finding suggested that, in addition to Prmt7, there is another mechanism that has a protective effect on cardiomyopathy in females. Therefore, we initially speculated that E2 could be the primary candidate for providing this protection. As the body ages, the balance between ROS production and antioxidant levels is generally dysregulated. E2 is involved in antioxidant defense mechanisms^20,26^. Thus, females are less susceptible to oxidative stress, which is associated with the stimulation of the production of proinflammatory cytokines, such as IL-1, IL-6, and TNFα, and is ultimately linked to various diseases, including cardiac disease^27,28^. As E2 becomes markedly reduced during postmenopause, the level of ROS increases, leading to an increase in oxidative stress and the inflammatory response. Therefore, we sought to determine the correlation between E2 and Prmt7 in oxidative stress and apoptosis in cardiomyocytes. Hereto, we found that E2 hardly alleviated the increase in DOX-mediated cardiotoxicity caused by the lack of Prmt7 activity, whereas the overexpression of Prmt7 enhanced the cardioprotective function of E2. Therefore, although Prmt7 is required for the full activation of E2, the complete interplay between E2 and Prmt7 in female cardiac protection is still unclear.

To identify additional candidates that are influenced by Prmt7 depletion under OVX conditions, we conducted RNA sequencing analysis of WT-OVX and cKO-OVX hearts. When Prmt7 was deficient in the heart, there were significant changes in the expression of genes related to immune responses (113 nodes), such as those related to the activation of the immune response, the production of TNF and IFN, and immune response-regulating signaling pathways (Fig. 6a and Supplementary Fig. 1). This finding indicates a strong association between the depletion of Prmt7 and increases in immune and inflammatory responses. Ultimately, we identified Socs3 as a candidate gene, and we observed that depletion of Prmt7 results in the repression of Socs3 expression, which leads to the activation of the JAK/STAT signaling pathway. According to these findings, we deduced the role of Prmt7 in the hearts of postmenopausal females (Fig. 7k). After menopause in females, a decrease in E2 levels is linked to an increase in oxidative stress and the inflammatory response, which are associated with the development of cardiomyopathy. Under these conditions, Prmt7 is involved in regulating the JAK/STAT signaling pathway by controlling Socs3 transcription, consequently diminishing inflammation and oxidative stress. However, if Prmt7 is depleted after menopause, STAT3 signaling is dysregulated due to decreased negative feedback by Socs3, eventually resulting in more severe cardiomyopathy. Thus, Prmt7 likely plays a role in the complete activation of E2-mediated cardioprotection by inhibiting inflammatory responses and oxidative stress.

The JAK/STAT signaling pathway has emerged as a key target for various diseases. In the heart, STATs regulate the expression of genes that encode proteins involved in inflammation, apoptosis, angiogenesis, and the ECM composition^29,30^. Activation of STAT-5A and STAT-6 stimulates the production of proinflammatory cytokines, thereby contributing to the pathogenesis of myocardial ischemia/reperfusion injury^31^. Additionally, activation of the JAK/STAT pathway leads to an imbalance between proinflammatory and anti-inflammatory cytokines, resulting in increased cardiovascular risks associated with rheumatoid arthritis and myeloproliferative neoplasms^32^.

According to studies conducted by various groups, arginine methylation by PRMTs is a crucial modification in regulating the JAK/STAT signaling pathway. Mowen et al. demonstrated that PRMT1 methylates Arg31 on STAT1, which is involved in IFN-α/β signaling activation. Arginine methylation of STAT1 suppresses the formation of a complex between STAT1 and PIAS1, an inhibitor of STAT1 dimer formation, thereby promoting STAT1-mediated IFN-α/β signaling activity^33^. This result was confirmed by liver biopsies from hepatitis C virus-infected patients, in which IFN signaling activity was attenuated and the level of arginine-methylated STAT1 was also reduced; in addition, the STAT-PIAS1 interaction was increased^34^, indicating that arginine methylation by PRMT1 is critical for regulating interferon-dependent cell responses through STAT1 methylation. STAT6 is also known to be methylated at Arg27, which is critical for its phosphorylation, nuclear translocation, and DNA binding activity. Interestingly, this modification is not associated with inhibiting the binding of PIAS^35^. STAT3 is required for leptin signaling, and arginine methylation of STAT3 by PRMT2 is necessary for the activation of STAT3-mediated leptin signaling^36^. Prmt5 was discovered to be a JAK2-binding protein. However, it has been found that JAK2 is not a substrate for methylation by Prmt5. Instead, Prmt5 methylates Smad7, which stimulates its interaction with gp130, an IL-6 coreceptor, eventually leading to the activation of STAT3^37^. Previously, CARM1 (PRMT4) was shown to contribute to the activation of several steroid-responsive genes after interacting with p/CIP (the p/300 CBP-interacting protein)^38^. Later, Coughlan et al. performed ChIP-on-ChIP analysis and genome-wide microarray analysis to search for promoter targets of the CARM1-p/CIP complex that are responsive to E2^39^. Among the targets, the CARM1-p/CIP complex is enriched in the *JAK2* promoter, which regulates E2-dependent JAK2 transcription and ultimately activates the JAK/STAT signaling pathway. In the present study, we demonstrated that Prmt7 is required for the expression of Socs3, which in turn can inhibit STAT activity, thereby controlling inflammation.

In conclusion, we provide evidence that Prmt7 attenuates menopause-associated oxidative stress and inflammation, which are strongly linked to severe cell death in female cardiomyocytes. Thus, these findings suggest that Prmt7 could be a plausible therapeutic molecule for managing menopause-associated heart failure.

### Supplementary information

Supplementary Figure
