# Supplementary material for: Prmt7 regulates the JAK/STAT/Socs3 signaling pathway in postmenopausal cardiomyopathy
Source: Exp Mol Med. 2024 Mar 14;56(3):711–20. doi: 10.1038/s12276-024-01193-3 (PMC10985114; doi:10.1038/s12276-024-01193-3)
Supplement: Supplementary file 1 — Supplementary Figure [file 12276_2024_1193_MOESM1_ESM.pdf]

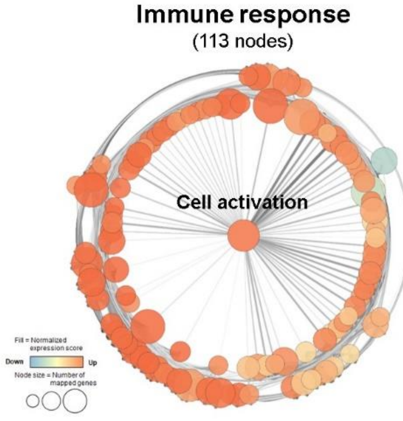

Representative GO terms	NES
Activation of immune response	1.9472
Innate immune response	1.9078
TNF cytokine production	1.8863
Immune response regulating signaling pathway	1.8825
Defense response to other organism	1.8647
Immune response regulating cell surface receptor signaling pathway	1.8281
Activation of innate immune response	1.8134
Immune effector process	1.6696
Interferon-6 production	1.5585
Cytokine mediated signaling pathway	1.5203

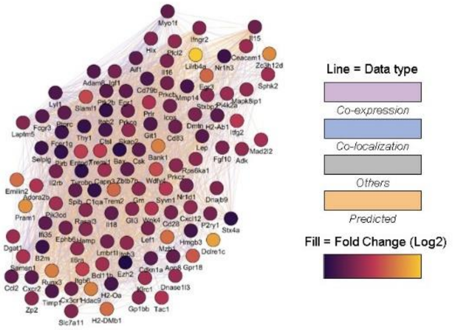

Supplementary Fig. 1. Prmt7-deficient hearts of OVX mice exhibit altered gene expression profiles associated with immune response

Enrichment map comparing gene ontology (GO) terms between OVX-WT and OVX-cKO mice. Nodes represent gene sets connected by edges, grouped in sub-clusters, and manually annotated. Gene interaction network was constructed from "immune response" gene sets, with different lines indicating interaction types. Fold-change (FC) > 1.3, normalized data (log2) > 1.0 and *P*-values cut-off < 0.1 using student's *t*-test. The genes were initially analyzed using GSEA, followed by an Enrichment map analysis using Cytoscape.
